# Subthalamic stimulation breaks the balance between distal and axial signs in Parkinson’s disease

**DOI:** 10.1038/s41598-021-01386-0

**Published:** 2021-11-08

**Authors:** Cyril Atkinson-Clement, Émilie Cavazzini, Alexandre Zénon, Thierry Legou, Tatiana Witjas, Frédérique Fluchère, Jean-Philippe Azulay, Christelle Baunez, Serge Pinto, Alexandre Eusebio

**Affiliations:** 1 Sorbonne Université, Institut du Cerveau - Paris Brain Institute - ICM, Inserm, CNRS, AP-HP, Hôpital de la Pitié Salpêtrière (DMU 6), Paris, France; 2 Aix-Marseille Univ, CRNS, LPL, Aix-en-Provence, France; 3 INCIA, Université de Bordeaux, CNRS UMR5287, Bordeaux, France; 4 Institut de Neurosciences de la Timone, UMR7289, CNRS and Aix-Marseille Univ, Marseille, France; 5 Department of Neurology and Movement Disorders, Aix-Marseille Univ, APHM, CHU Timone, Marseille, France; 6 Aix-Marseille Univ, CNRS, LNC, Marseille, France

**Keywords:** Parkinson's disease, Motivation, Reward, Basal ganglia

## Abstract

In Parkinson’s disease (PD), the effects of both L_dopa_ and subthalamic deep brain stimulation (STN-DBS) are known to change cost-valuation. However, this was mostly studied through reward-effort task involving distal movements, while axial effort, less responsive to treatments, have been barely studied. Thus, our objective was to compare the influence of both L_dopa_ and STN-DBS on cost-valuation between two efforts modalities: vowel production (as an example of axial movement) and hand squeezing (as an example of distal movement). Twelve PD patients were recruited to participate in this study. The task consisted in deciding whether to accept or reject trials based on a reward-effort trade-off. Participants performed two blocks with hand squeezing, and two with vowel production, in the four treatment conditions (L_dopa_ *On*/*Off*; STN-DBS *On*/*Off*). We found that STN-DBS changed the ratio difference between hand and phonation efforts. Vowel production effort was estimated easier to perform with STN-DBS alone, and harder when associated with L_dopa_. The difference between hand and phonation efforts was correlated with quality of life in *Off*/*Off* and *On* L_dopa_ alone conditions, and with impulsive assessment *On* STN-DBS alone. We highlighted that STN-DBS could introduce an imbalance between the actual motor impairments and their subjective costs. With this finding, we also suggest paying particular attention to the different treatment effects that should be expected for axial and distal movement dysfunctions.

## Introduction

Parkinson’s disease (PD) is a neurodegenerative disease associated to motor and cognitive impairments. Recent studies explored motivation deficits and treatment effects in PD by using motor effort-based decision-making tasks^1^. These studies reported that PD patients show over-valuation of handgrip effort compared to healthy controls (HC), which can be normalized^2^ or not^3^ under medication. A ventral striatal dysfunction^2^ has been suggested to explain this motivation deficit in PD, which is in coherence with the hypothesis of mesolimbic (involved in reward valuation^4,5^) and mesocortical (involved in effort processing^6,7^) system impairments^8^. Subthalamic deep brain stimulation (STN-DBS), was shown to normalise effort-based decision-making in PD^3^, in agreement with our previous research highlighting that STN is involved in cost–benefit valuation^9^. In fact, among PD symptoms, altered distal hand movements are the most responsive to pharmacological and neurosurgical treatments.

However, this is less the case with axial signs, including speech impairment^10–12^. In detail, L_dopa_ can improve some dimensions of speech such as vocal loudness^13,14^, but not intelligibility^12,15^. STN-DBS can improve articulation features but might alter voice and respiratory functions^16^. Vowel production is often assessed in PD patients, as a single phonatory production that can address several information on laryngeal dysfunction; it can be considered as a marker of PD progression^17^ and reliable to study articulatory dysfunction and treatment effects such as STN-DBS^18^.

Due to their specific pathophysiologies and treatment responses different from those of more lateralized movements, we hypothesize that treatments (i.e., L_dopa_ and STN-DBS) would also change differently the subjective cost-valuation of axial and distal efforts in PD. To compare axial and lateralized cost-valuations, we focused on the two following modalities: vowel production—a sub-component of phonation—as an example of axial function, and hand squeezing as an example of distal movement. In other words, could vowel production be perceived over-costly in PD in comparison to hand squeezing, leading patients to engage less in speech production?

## Methods

### Patients

Twelve patients (10 males and 2 females) suffering from idiopathic PD, fulfilling UK Parkinson’s Disease Brain Bank Criteria^19^, and operated bilaterally for STN-DBS were recruited in the Department of Neurology and Movement Disorders of La Timone University Hospital (Marseille, France), after providing written informed consent for themselves. This project was approved by the local ethics committee (CPP Sud-Méditerranée) and realized in compliance with the national legislation and the Declaration of Helsinki^20^. All patients underwent a bilateral implantation of stimulation electrodes within the STN. The features of the electrodes (Medtronic, model 3389) were the following: 4 contacts, 1.27 mm diameter and 1.5 mm height, separated by insulating bands of 0.5 mm height.

All participants were screened, under L_dopa_ and STN-DBS turned *On*, for depression (Beck Depression Inventory [BDI^21^]), anxiety (Hamilton Anxiety Rating Scale [HAM-A^22^]), impulsivity (Barratt Impulsiveness Scale [BIS-11^23^]; Questionnaire for Impulsive-Compulsive Disorders [QUIP^24^]; Sensitivity to Punishment and Sensitivity to Reward Questionnaire [SPSRQ^25^]), speech impairment (Voice Handicap Index [VHI^26^]; Dysarthria Impact Profile [DIP^27,28^]), and quality of life (Parkinson’s Disease Questionnaire–39 [PDQ-39^29^]). Motor impairment was assessed under four treatment conditions (without treatment [*Off* Dopa/*Off* DBS], with L_dopa_ alone [*On* Dopa/*Off* DBS], with STN-DBS alone [*Off* Dopa/*On* DBS], with both treatments [*On* Dopa/*On* DBS]), using the Unified Parkinson’s Disease Rating Scale part-III (UPDRS-III^30^). Demographics and clinical characteristics of the patients are presented in Table 1.

**Table 1:** Demographics, clinical data and STN-DBS parameters of the Parkinson’s disease patients.

	Mean ± SD	[Min–Max]
Age	60.8 ± 8.1	[45–75]
Disease duration (years)	13.2 ± 6.9	[6–31]
**UPDRS**–**III**–**Total**		
*Off* Dopa/*Off* DBS	30.5 ± 13.6	[9–49]
*On* Dopa/*Off* DBS	11.3 ± 9.1	[1–23]
*Off* Dopa/*On* DBS	12.6 ± 7.9	[3–29]
*On* Dopa/*On* DBS	5.6 ± 5.5	[0–18]
**UPDRS**–**III**–**Speech**		
*Off* Dopa/*Off* DBS	0.89 ± 0.6	[0–2]
*On* Dopa/*Off* DBS	0.6 ± 0.5	[0–1]
*Off* Dopa/*On* DBS	0.73 ± 0.4	[0–2]
*On* Dopa/*On* DBS	0.55 + 0.7	[0–2]
Hoehn & Yahr	2.2 ± 0.6	[1–3]
DBS duration (months)	11.5 ± 8.4	[6–36]
DBS–L–Hz	139.1 ± 20.2	[120–190]
DBS–R–Hz	139.1 ± 20.2	[120–190]
DBS–L–V	2.2 ± 0.5	[1.2–3]
DBS–R–V	2.1 ± 0.5	[1.1–3]
LED (mg)	250 ± 52.7	[150–300]
BDI	7.1 ± 3.5	[3–14]
HAM-A	6.6 ± 6.5	[0–20]
BIS-11	65.6 ± 11.7	[50–88]
QUIP	1.9 ± 3.8	[0–12]
SPSRQ	66.6 ± 16.8	[41–92]
VHI	28 ± 23.1	[0–77]
DIP	172.1 ± 17.9	[142–206]
PDQ-39	48.4 ± 18.9	[14–82]

BDI: Beck Depression Inventory; BIS-11: Barratt Impulsiveness Scale; DIP: Dysarthria Impact Profile; HAM-A: Hamilton Anxiety Rating Scale; Hz: STN-DBS frequency in hertz; LED: levodopa equivalent dose during the experiment (Tomlinson et al., 2010); PDQ-39: Parkinson’s Disease Questionnaire–39; QUIP: Questionnaire for Impulsive-Compulsive Disorders; SPSRQ: Sensitivity to Punishment and Sensitivity to Reward Questionnaire; UPDRS: Unified Parkinson’s Disease Rating Scale; V: STN-DBS voltage in volts; VHI: Voice Handicap Index.

### Task and procedure

The experimental task was an adapted version of the one previously used and described^3,9^. Before starting, participants were trained with a slowed-down version of the task, allowing the experimenter to explain the meaning of each phase of the task and the responses that were expected from them.

After the patients were comfortably seated, they had to perform firstly a “maximal effort” during 7.6 s, repeated three times. This maximal effort consisted in squeezing a dynamometer with their dominant hand as strongly as possible (the “*hand maximal effort*”) or to sustain the vowel /a/ at loud and comfortable (not forced) volume (the “*phonation maximal effort*”). Participants had to perform these maximal effort tasks before each experimental block (i.e., 4 times). Maximal efforts corresponded to the integral of exerted force over time (i.e., the total of pressure exerted during the 7.6 s) and were used as a reference for the rest of the protocol to adapt difficulty for each participant and each block Secondly, each patient actually performed the task, split in 16 blocks of eight minutes for a total duration of about two hours: four blocks for each treatment conditions (*Off* Dopa/*Off* DBS; *On* Dopa/*Off* DBS; *Off* Dopa/*On* DBS; *On* Dopa/*On* DBS) and within these treatment conditions, twice for hand squeezing, and twice for vowel production.

Each trial started with a fixation cross displayed for 500-1500 ms. Then, the virtual monetary reward value of the trial was displayed (3 rewards: 5c, 20c, 100c). A second cue indicated the level of effort required for the current trial to obtain the reward, represented as a vertical gauge with a horizontal bar indicating the level of effort duration. These effort levels corresponded to the application of the previously measured “maximal effort” during 1.26 s (level 1), 2.53 s (level 2), 3.8 s (level 3), 5.06 s (level 4), 6.33 (level 5) or 7.6 s (level 6).

At this time, the patients were instructed to decide whether they accepted the trial. In other words, they had to decide whether or not the promised reward was worth performing the required effort. If they accepted, they had to start squeezing the dynamometer or producing the vowel. Subjects were free to vary how these effort levels were achieved by trading duration for force. In other words, if the subjects applied a lower force than “maximal effort”, the gauge completion increased slowly, whereas if they applied a higher force the gauge completion increased quickly. Therefore, a short effort duration reflected a higher force applied while a longer effort related to a lower force applied.

**Figure 1:**
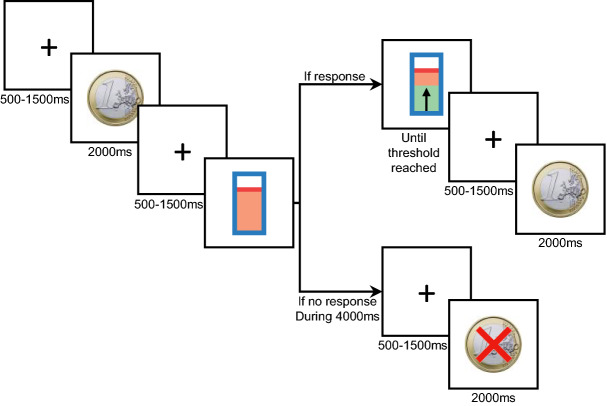
Experimental design of the study.

When the level indicated on the gauge reached the threshold, a fixation cross was displayed for 500-1500 ms, followed by the reward feedback, accompanied by a bell sound, shown on the screen for 2000 ms (Fig. 1). Any effort produced before the effort cue onset stopped the trial. When the patients wished to refuse the proposed trial, they had to withhold their response for 4 s, then a display of the reward amount they had refused was shown superimposed with a red crossed on the screen for 2000 ms. Intercue intervals were randomized to limit the expectation of the upcoming cue and any related preparation. Each of the 18 conditions (3 reward possibilities × 6 effort levels) were presented in randomized order.

The task was performed on two separate mornings (one half-day *Off* L_dopa_, the other *On* L_dopa_). The *Off* L_dopa_ sessions were realized after an overnight (12 h) withdrawal of treatment^31^. The *On* L_dopa_ sessions were performed 45–60 min after the intake of a unique L_dopa_ dose corresponding to 150% of each patient’s usual morning dose. The *Off*/*On* STN-DBS sessions were carried out at least 20 min after STN-DBS switched *Off*/*On.* The treatment conditions were applied in a pseudo-randomized order.

### Outcome measures and statistical analyses

Five outcome measures were analysed. The “maximal efforts” which were analysed separately for vowel production and hand squeezing in order to identify treatment or block order influences. Then, we analysed the probability of accepting trials (i.e., acceptance rate), decision time to accept trials (i.e., the time before starting the effort) and the applied force (i.e., the effort duration which reflects the force applied). Last, we analysed the difference of acceptance rate between hand squeezing and vowel production efforts, which was computed separately for each treatment condition and for each participant. A positive difference indicated a preference for choosing hand squeezing while a negative one reflected the preference of choosing vowel production.

All statistical analyses were conducted with R^33^. Statistical analyses consisted of generalised linear mixed models followed by Tukey post-hoc tests to correct for multiple comparisons and bootstrapped (n = 10,000) Pearson’s correlations to capture the 95% highest density interval (95HDI) of the correlation coefficients. For mixed models, a threshold of significance was set at *p* ≤ 0.05. For correlations, we considered as significant if the lower and upper values of the 95% highest density interval (95HDI) were both higher or below 0.

## Results

Efficacy of the treatment conditions was confirmed using the UPDRS-III score. L_dopa_ administration leads to an improvement of 69.1% ± 21.8% compared with the *Off*-drug state. STN-DBS leads to an improvement of about 75%^32^.

### Maximal effort

Maximal effort was not influenced by block order for both hand squeezing (F_(1;96)_ = 3.69; *p* = 0.058; *d* = 0.43) and vowel production (F_(1;96)_ = 1.39; *p* = 0.24; *d* = 0.262). However, hand squeezing maximal effort was significantly higher in the *Off* Dopa/*On* DBS condition in comparison to the *Off*/*Off* condition (main effect: F_(3;96)_ = 2.91; *p* = 0.039; *d* = 0.66; post-hoc: *p* = 0.035), which was not observed for vowel production maximal effort (F_(3;96)_ = 1.73; *p* = 0.17; *d* = 0.5).

### Acceptance rate

**Figure 2:**
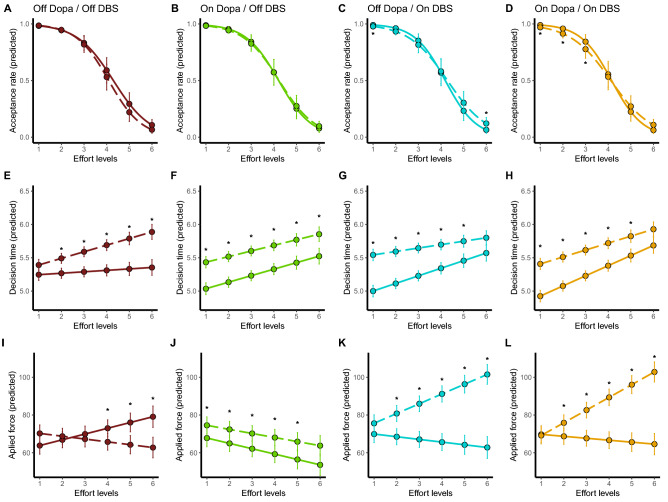
Acceptance rate (part **A** to part **D**), decision time (parts **E** to part **H**) and applied force (part **I** to **L**) per treatment, efforts nature and efforts levels. Solid lines represent hand efforts and dashed lines represent phonation efforts. The applied force corresponds to the percentage of maximal effort performed during the calibration session.

We found significant main effects of both effort (F_(1;5076)_ = 1115.47; *p* < 0.0001; *d* = 0.93) and reward levels (F_(1;5076)_ = 72.41; *p* < 0.0001); *d* = 0.24) but no main effect of effort nature (F_(1;5076)_ = 1.3; *p* = 0.26; *d* = 0.03) or treatment condition (F_(1;5076)_ = 0.66; *p* = 0.56; *d* = 0.07). However, we found a significant interaction between effort level and nature (F_(1;5076)_ = 6.37; *p* = 0.0149; *d* = 0.07) and between effort level, nature and treatment condition (F_(3;5076)_ = 3.25; *p* = 0.0207; *d* = 0.09; Fig. 2A–D). More in detail, Tukey post-hoc tests allowed to determine that acceptance rate was (i) lower for vowel production than hand squeezing for the lowest effort level (i.e., level 1: *p* = 0.032) and for the highest effort level (i.e., level 6: *p* = 0.037) during the *Off* Dopa/*On* DBS condition; (ii) lower for vowel production than for hand squeezing during the low effort levels (level 1: *p* = 0.004; level 2: *p* = 0.006; level 3: *p* = 0.032) during the *On* L_dopa_/*On* DBS condition.

### Decision time

Regarding decision time, main effects were found for the effort level (F_(1;2438)_ = 90.83; *p* < 0.0001; *d* = 0.37), the reward level (F_(1;2438)_ = 19.34; *p* < 0.0001; *d* = 0.18) and the effort nature (F_(1;2438)_ = 39.42; *p* < 0.0001; *d* = 0.25) but not for the treatment condition (F_(1;2438)_ = 3.16; *p* = 0.67; *d* = 0.12). In addition, an interaction between the effort level, effort nature and the treatment condition was significant (F_(1;2438)_ = 3.04; *p* = 0.028; *d* = 0.12; Fig. 2E–H). Tukey post-hoc tests showed significant differences between vowel production and hand squeezing, in all treatment conditions, but not for the lowest effort level when patients were in the Off/Off condition and for the highest effort level when patients were under STN-DBS alone or combined with medication (*p* < 0.05).

### Applied force

We also observed that the applied force was significantly influenced by the effort level (F_(1;2438)_ = 6.67; *p* = 0.009; *d* = 0.1), the reward level (F_(1;2438)_ = 4.57; *p* = 0.032; *d* = 0.09), the effort nature (F_(1;2438)_ = 68.23; *p* < 0.0001; *d* = 0.33) and the treatment condition (F_(3;2438)_ = 18.95; *p* < 0.0001; *d* = 0.3). In addition, Tukey post-hoc tests revealed that all interactions were significant except those with the reward level. In particular (Fig. 2I–L), the applied force was higher for vowel production in comparison to hand squeezing, except during the Off/Off condition.

### Correlations with clinical variables

Firstly, none of the correlations between clinical variables and the difference of hand squeezing vs. vowel production acceptance rate reached the threshold of significance, whatever the treatment condition (*p* > 0.09; *d* < 1.178).

**Figure 3:**
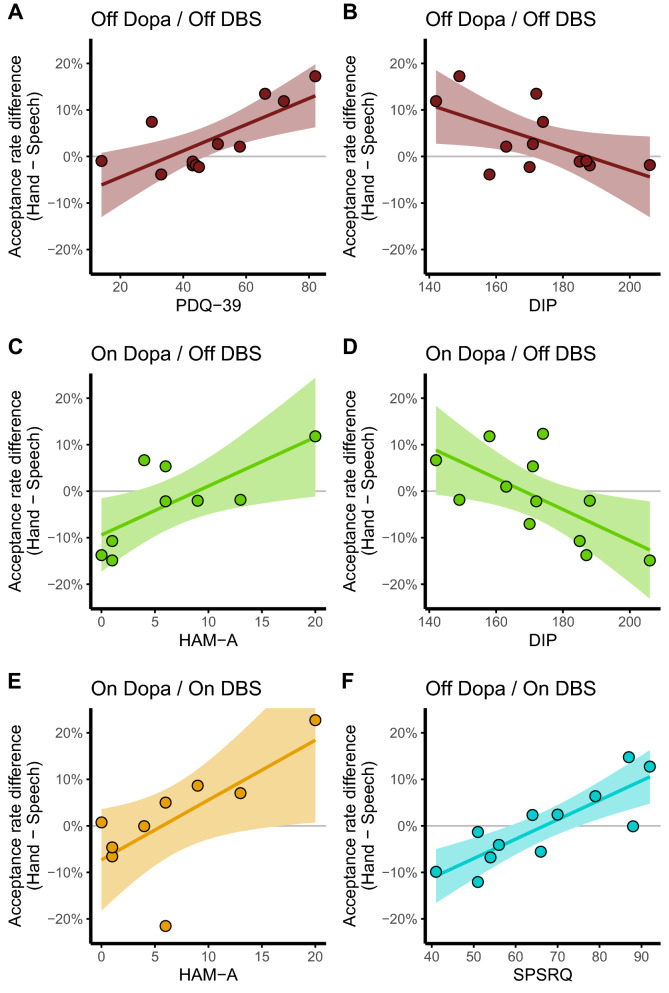
Significant correlations between the difference of acceptance rate between hand and phonation per treatment conditions. A positive value indicates a preference for a hand effort, a negative value indicates a preference for a speech effort, and null value indicates an absence of difference between a hand and a speech effort. DIP: Dysarthria Impact Profile; HAM-A: Hamilton Anxiety Rating Scale; PDQ-39: Parkinson’s Disease Questionnaire–39; SPSRQ: Sensitivity to Punishment and Sensitivity to Reward Questionnaire.

However, we found that the acceptance rate difference in the *Off*/*Off* condition was related to quality of life (total PDQ-39 score, F_(1;10)_ = 12.53; *p* = 0.005; *d* = 2.238; r(95HDI) = [0.44;0.95]; Fig. 3A) and the psychosocial impact of dysarthria (total DIP score, F_(1;10)_ = 5.25; *p* = 0.045; *d* = 1.45; r(95HDI) = [− 0.86; − 0.22]; Fig. 3B). The correlation with the PDQ-39 was mainly driven by the subscales of social support (F_(1;9)_ = 41.83; *p* < 0.0001; *d* = 4.312; r(95HDI) = [0.76;0.96]), communication (F_(1;9)_ = 10.05; *p* = 0.011; *d* = 2.112; r(95HDI) = [0.52;0.91]), activities of daily living (F_(1;9)_ = 8.15; *p* = 0.019; *d* = 1.904; r(95HDI) = [0.55;0.92]) and cognition (F_(1;9)_ = 6.1; *p* = 0.035; *d* = 1.648; r(95HDI) = [0.44;0.82]). The acceptance rate difference in the *On* Dopa/*Off* DBS condition was correlated to anxiety (HAM-A, F_(1;7)_ = 8.18; *p* = 0.024; *d* = 2.162; r(95HDI) = [0.42;0.96]; Fig. 3C) and the psychosocial impact of dysarthria (DIP total score, F_(1;10)_ = 7.37; *p* = 0.021; *d* = 1.718; r(95HDI) = [− 0.88; − 0.36]; Fig. 3D). *On* Dopa/*On* DBS, it was correlated only to anxiety (HAM-A, F_(1;7)_ = 6.38; *p* = 0.039; *d* = 1.908; r(95HDI) = [0.3;0.97]; Fig. 3E). The acceptance rate difference *Off* Dopa/*On* DBS was correlated only to the sensitivity to punishment and reward (SPSRQ, F_(1;10)_ = 23.75; *p* = 0.0006; *d* = 3.082; r(95HDI) = [0.67;0.96]; Fig. 3F). No significant correlation was found with the UPDRS-III total score (*p* > 0.062; *d* < 1.53).

## Discussion

To the best of our knowledge, this study is the first to compare the effects of both L_dopa_ and STN-DBS on subjective effort-valuation for two motor modalities: hand squeezing and vowel production. As expected, we found that treatment combinations have different effects on the subjective valuation for these two effort modalities, supported also by different correlations between clinical scores and experimental measures. In more details, the *Off*/*Off* and the *On* Dopa/*Off* DBS conditions were characterised by an absence of difference between hand efforts and vowel production regarding acceptance rate, but we found that the more the patients avoided vowel efforts, the more they had a decreased quality of life related to speech impairment (i.e., PDQ and DIP). Acceptance rate differences were exclusively found during the conditions which involved STN-DBS, with a decreased vowel effort acceptance rate for the low efforts and an increased vowel effort acceptance rate for high efforts during the *On*/*On* condition in comparison to hand effort. Interestingly, the difference of acceptance rate between the two effort modalities was correlated to a measure of punishment and reward sensitivity during the *Off* Dopa/*On* DBS condition (i.e., SPSRQ) and with a measure of anxiety during the *On*/*On* condition.

For the subjective cost of vowel production in comparison to hand squeezing, we found no difference of acceptance rate in the *Off*/*Off* condition, while decision time and applied force findings suggest that vowel production was harder than hand efforts. The difference between the hand squeezing and vowel production acceptance rates was related to the quality of life, mainly driven by communication PDQ-39 items. The higher the cost of vowel production relative to hand squeezing, the lower was the quality of life of patients. This result is even more interesting as we found no association between the acceptance rate difference and the motor impairment assessed by the UPDRS part III. It suggests that the decrease of quality of life, and mainly communication deficits, could be associated to over-valuation of phonation cost. This finding is also in agreement with the notion of functional prioritization of one action (e.g., hand movement or squeezing) to others (e.g., phonation of speech productions)^34^, also observed with the “stop walking when talking” effect^35^.

During the L_dopa_ alone condition, we found (i) no difference of acceptance rate between hand squeezing and vowel production, (ii) no slopes difference on the decision time and the applied force, and (iii) a similar correlation between the acceptance rate difference and quality of life observed in the *Off*/*Off* condition. This lack of L_dopa_ modulation across effectors is not surprising, since our task involved a simple sustained vowel production, which is restrictive to a phonatory task. Phonatory function was already found improved by L_dopa_ in PD^36^. Thus, one can imagine that L_dopa_ modulated in the same way hand squeezing and vowel production cost-valuations. This hypothesis is reinforced by the observation of the same correlation during the *Off*/*Off* and *On* Dopa/*Off* DBS conditions between the difference of acceptance rate and the quality of life related to communication (i.e., DIP). Moreover, our result reinforces previous observations using cost-valuation for hand efforts which suggested a limited effect of L_dopa_^3,37,38^, which is more related to reward-valuation^6^.

Different effects were obtained by STN-DBS on hand and vowel production efforts. Under STN-DBS alone, only for the highest effort level, a preference for vowel production in comparison to hand squeezing was found, meaning that vowel production was perceived easier to perform than hand squeezing for this effort level. Moreover, this difference was correlated to the degree of sensitivity for reward and punishment (i.e., SPSRQ), known to be related to inhibition impairment, risky behaviours, extraversion and impulsivity^25^. This is most likely since axial signs in PD, including laryngeal dysfunction^39,40^ involved in vowel production, evolve in parallel to cognitive impairments and higher risk of impulsive disorders. Also, STN-DBS could have a general positive influence on Parkinsonian signs, including axial ones, by increasing motivation (i.e., higher acceptance rate than during the *Off*/*Off* state, see^3^) and decreasing vowel production subjective cost. This is of importance since the effect of STN-DBS on decision-making processes and impulsive behaviours represents an ongoing matter of debate.

Conversely, during the condition combining STN-DBS with L_dopa_, we mainly observed a decreased acceptance rate for vowel production in comparison to hand squeezing for the lowest efforts levels, meaning that vowel production was considered more effortful than hand squeezing. This would suggest a negative synergistic effect of L_dopa_ and STN-DBS on the subjective cost of vowel production. If the studies that compare medication and functional neurosurgery on dysarthria in PD are rare, it was reported that L_dopa_ associated to STN-DBS could impair vowel production^41^, in comparison to single treatment conditions, i.e. STN-DBS alone or L_dopa_ alone. In the same conceptual vein, one animal study also suggested that STN-DBS could modulate dopaminergic releasing^42^. Therefore, in this treatment condition, one can imagine that, in comparison to lateralized movements, vowel production, as an indirect reflect of laryngeal axial function, could be (at least partly) the result of both a higher-cognitive cost over-valuation and a motor impairment.

Our main finding suggests that STN-DBS could break the balance between the subjective cost-valuation of distal (i.e., hand squeezing) and axial (i.e., vowel production) efforts. In other words, even if both medication and STN-DBS have no implication on the ability to perform a high effort during a short time (i.e., maximal efforts), they influence the cost-representation of these actions. To go further, we hypothesize that vowel production impairment in PD implies subjective cost over-valuation of the actual impairment, especially when STN-DBS is turned *On* and associated with L-dopa. This is also supported by the fact that the subjective cost of vowel production was not in coherence with clinical outcomes such as the UPDRS part III and the maximal efforts. In fact, association of a speech task to another task was found to impair speech during complex (i.e., double tasks;^43–45^) and simple contexts (i.e., combined tasks;^46^). Also, one interesting study compared overt to covert speech production in PD, with and without medication^47^. The authors found that overt and covert speech were associated with abnormal brain activations in PD, normalised under medication for overt speech only. Medication would improve motor speech performance, but not non-motor aspects.

We have to acknowledge several limitations to our study. The first one is related to the participants recruited. The sample size, composed of twelve PD patients, is low and heterogeneous. This limitation was due to our methodological objective, which implied that all included patients performed the task under the four treatments conditions, instead of recruiting several groups of different participants. Also, an additional group of age-matched elderly healthy controls could have been considered, especially to determine if the patients’ behaviours were outside the normal range. Future studies should have a higher number of participants, a better group homogeneity and an additional group of healthy controls. The second one is more related to the tasks. The experiment was particularly long for the patients (i.e., eight blocks per day on two half-days, without optimal treatments) and implied two efforts modalities as examples of distal and axial PD’s symptoms. In addition, we chose to use the dominant hand rather than, systematically, the hand with dominant PD symptoms. Second step studies would complement our results with additional testing of efforts of other natures and by comparing the most and less affected sides.

## Conclusion

Our results highlighted that both medication and STN-DBS could modify the subjective cost of axial (i.e., vowel production) and distal (i.e., hand squeezing) efforts differently. Therefore, these treatments could introduce an imbalance between the actual motor impairments and their subjective cost, according to their lateralized or axial dimensions.

## Data Availability

The datasets generated and analysed during the current study are available from the corresponding author on reasonable request.
